# Evolution of Mutational Robustness in an RNA Virus

**DOI:** 10.1371/journal.pbio.0030381

**Published:** 2005-11-01

**Authors:** Rebecca Montville, Remy Froissart, Susanna K Remold, Olivier Tenaillon, Paul E Turner

**Affiliations:** **1** Department of Ecology and Evolutionary Biology, Yale University, New Haven, Connecticut, United States of America,; **2** INSERM, Equipe, Ecologie et Evolution de Micro-Organismes, Henri Huchard, Paris, France; Massey UniversityNew Zealand

## Abstract

Mutational (genetic) robustness is phenotypic constancy in the face of mutational changes to the genome. Robustness is critical to the understanding of evolution because phenotypically expressed genetic variation is the fuel of natural selection. Nonetheless, the evidence for adaptive evolution of mutational robustness in biological populations is controversial. Robustness should be selectively favored when mutation rates are high, a common feature of RNA viruses. However, selection for robustness may be relaxed under virus co-infection because complementation between virus genotypes can buffer mutational effects. We therefore hypothesized that selection for genetic robustness in viruses will be weakened with increasing frequency of co-infection. To test this idea, we used populations of RNA phage φ6 that were experimentally evolved at low and high levels of co-infection and subjected lineages of these viruses to mutation accumulation through population bottlenecking. The data demonstrate that viruses evolved under high co-infection show relatively greater mean magnitude and variance in the fitness changes generated by addition of random mutations, confirming our hypothesis that they experience weakened selection for robustness. Our study further suggests that co-infection of host cells may be advantageous to RNA viruses only in the short term. In addition, we observed higher mutation frequencies in the more robust viruses, indicating that evolution of robustness might foster less-accurate genome replication in RNA viruses.

## Abstract 2

RNA phage viruses evolved under high co-infection of host cells are less robust to mutations than those propagated under low co-infection, suggesting that co-infection may be advantageous to RNA viruses only in the short term.

## Introduction

Mutational (genetic) robustness can be defined as constancy of phenotype in the face of mutational perturbation [1]. Genetic variance and differences in phenotypic performance among genotypes underlie all of Darwinian evolution. Thus, robustness is crucial to the understanding of evolution because it dictates phenotypic expression of genetic variation [2]. But it remains unclear whether robustness is merely accidental or a consequence of natural selection. The most straightforward explanation for the evolution of robustness is adaptationist. For a well-adapted population, almost all mutations lead to deviations from optimal performance in the selective environment. Populations at equilibrium should therefore experience selection for mutational robustness. However, evolution of genetic robustness is hard to observe in most laboratory systems because equilibrium states are difficult to achieve (or definitively prove) and the benefit of mutational robustness is not experienced until offspring carrying mutations arise [2]. For these reasons, the evidence for adaptive evolution of mutational robustness in biological populations remains controversial [3–5]. Therefore, the vast majority of studies demonstrating the phenomenon have relied on theory [6] or artificial life systems [7].

High mutation rate is perhaps the most important prerequisite for adaptive genetic robustness [2], so mutational robustness should be strongly selected in biological systems experiencing elevated mutation rates [6]. Thus, strong candidates for observing adaptive robustness would be RNA viruses, which typically feature mutation rates that are orders of magnitude higher than in DNA systems [8]. In general, the theoretical predictions for adaptive robustness under elevated mutation rates assume that phenotype expression results solely from the underlying genotype. However, many viruses feature complementation, a mechanism whereby low-fitness genotypes can phenotypically profit from intracellular proteins made by co-infecting strains of high fitness [9–11]. Co-infection coupled with complementation can therefore act as a mechanism that provides phenotypic buffering in the event of genomic mutations, similar to other buffering mechanisms such as gene duplication and diploidy that might have evolved to facilitate canalization in higher organisms [12,13]. We recently demonstrated that complementation can buffer the harmful fitness effects of deleterious alleles in co-infecting populations of the segmented RNA phage φ6 [10]. Here we further examine the evolutionary consequences of virus co-infection, by predicting that co-infecting phages are likely to experience weakened selection for mutational robustness.

We previously conducted laboratory experimental evolution of φ6 populations at low and high levels of co-infection [14]. In that study, a single clone of the wild-type virus was used to found six replicate populations, which underwent adaptation to *Pseudomonas syringae* pathovar (pv) *phaseolicola* bacteria for hundreds of virus generations. Three of the populations were evolved at low multiplicity of infection (MOI; ratio of infecting viruses to bacterial cells), and three at high MOI. Co-infection level was controlled by mixing viruses and bacteria in liquid medium at a given MOI, allowing sufficient time for virus attachment to cells, and then plating a dilution of the mixture onto agar with a superabundance of cells. During overnight incubation, viruses formed distinct plaques in the lawn, which resulted from lysis of infected cells and the release of viral progeny that infected neighboring cells. The passage cycle was repeated by harvesting plaques, removing the bacteria by filtration, and mixing viruses and naive bacteria at the controlled MOI. A total of 60 passage cycles were conducted, which equaled roughly 300 viral generations (i.e., five generations occur during overnight plaque formation [14]). Every fifth generation, populations in the low co-infection treatment experienced MOI = 0.002 whereas those in the high co-infection treatment experienced MOI = 5; otherwise, all aspects of the treatment environments were equal. Assuming Poisson sampling [15], the proportion of cells infected with *n* phages is *P(n)* = e^−MOI^ × MOI. Therefore, at MOI = 0.002, only approximately 0.1% of all infected (*n* > 0) cells are co-infected and clonal infections predominate. In contrast, approximately 97% of infected cells should be multiply infected at MOI = 5, and co-infection predominates, generally with two to three viruses (the limit to co-infection in φ6 [16]) infecting each host. (The percentage difference in multiply-infected cells across the MOI treatments is probably less than 1,000-fold, because we observe greater than Poisson-expected entry of φ6 particles into the same cell when viruses are grown at low MOI [16].) Thus, the treatment environments were the same, except that populations at high MOI more often experienced co-infection, an environment demonstrated to allow intracellular virus interactions such as complementation [10].

Here we examined whether evolution of mutational robustness occurs differently for viruses evolved at low and high levels of co-infection. We tested the hypothesis by randomly isolating clones from each of the six previously evolved populations, and using these clones to found lineages that were subjected to a mutation accumulation experiment [17–19]. Mutation accumulation was achieved by serially propagating the lineages in a new environment where they experienced severe bottlenecking. The sampling of mutations in these experiments is nearly unbiased because genetic drift overwhelms natural selection during the extreme bottlenecks. By removing selection, all non-lethal mutations can increase to fixation with roughly the same probability, regardless of whether they are deleterious, advantageous, or neutral [17–19]. However, because most mutations are deleterious, mutation accumulation experiments tend to cause reduced fitness [17–19]. We compared the fitness consequences of mutation accumulation for lineages drawn from the different co-infection treatments, by measuring the mean magnitude and variance in fitness change that occurred as a result of bottlenecking. Our data confirmed the hypothesis that viruses historically evolved under high co-infection are relatively less robust than those evolved under low co-infection, demonstrated by their greater mean magnitude and variance in fitness changes generated by addition of random non-lethal mutations.

## Results

We isolated ten clones at random from each replicate population in the low co-infection and high co-infection level treatments at generation 300, and used these to found 60 independent virus lineages (ten clones × three populations × two co-infection treatments). We then conducted a mutation accumulation experiment [17,19], in which the lineages were subjected to a new habitat containing extreme population bottlenecks consisting of single-virus passages on *P. phaseolicola* for 20 d (see Materials and Methods). Plaque formation (i.e., five generations of ordinary virus growth) occurred in between bottlenecks, resulting in approximately 100 generations of virus evolution via mutation accumulation. Under these conditions, lineages may become fixed for any non-lethal mutation through the process of genetic drift. The genomic mutation rate in φ6 is gauged to be 0.067 deleterious mutations per generation [19]. We therefore estimated that one mutation on average had been fixed in each lineage (i.e., 0.067 × 20 bottleneck events ≍ 1.3), in which it is assumed that the majority of spontaneous mutations are deleterious. At the end of the mutation accumulation experiment, we conducted replicated (*n* = 3) fitness assays against a common virus competitor for the focal genotypes (pre-bottleneck founding clone and post-bottleneck final clone) of each lineage. In this way, we were able to measure the mean change in log_10_ fitness (Δlog_10_ *W*) for each lineage as a result of mutation accumulation; Δlog_10_ *W* = log_10_ *W*_post-bottleneck_ − log_10_ *W*_pre-bottleneck_. This design resulted in 360 total fitness estimates (60 lineages × two focal genotypes per lineage × three replicate estimates), in which focal genotypes of a lineage were always assayed within the same temporal block.

The Δlog_10_ *W* values estimate the fitness effect of adding roughly one random non-lethal mutation to the founding genotype of a lineage. Mutational robustness is defined as decreased phenotypic variability in the face of mutational change. Thus, one set of genotypes can be considered more robust than another collection of genotypes if the first group has a significantly lower variance in the fitness change brought on by addition of mutation(s) to the genome [2,20]. In our study, greater robustness would be indicated by relatively lower among-lineage variance in the fitness changes brought on by the bottlenecking experiment. We therefore analyzed the Δlog_10_ *W* values using mixed linear models that can fit different variances for random factors, depending on the level of the fixed factor with which they are associated [21]. Our approach is uniquely powerful for testing effects of co-infection treatment on the evolution of robustness, because we are able to estimate and test differences between the among-lineage variance for lineages drawn from low versus high co-infection level populations.

**Figure 1:**
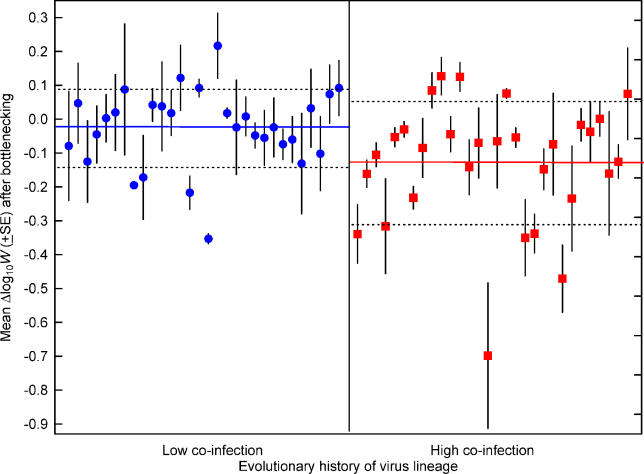
Viruses Evolved under Low Co-Infection Are More Robust than Those Evolved under High Co-Infection Each point is the mean (*n* = 3, ± standard error of the mean) change in log_10_ fitness (Δlog_10_ *W*) resulting from mutation accumulation, for an independent lineage founded by a virus clone evolved under low level of co-infection (circles) or high level of co-infection (squares). Horizontal lines are grand means for Δlog_10_ *W* among lineages within a treatment, and the dashed lines indicate one standard deviation away from the grand mean.

We detected significantly lower variance in Δlog_10_ *W* among the lineages evolved under low co-infection (Table 1); these data supported the hypothesis that selection for mutational robustness is stronger when viruses rarely experience co-infection. The relatively greater robustness of the low co-infection–evolved viruses is evident in Figure 1, which shows the tighter clumping of mean Δlog_10_ *W* values around the grand mean for virus lineages evolved under low co-infection, and the lower deviation in Δlog_10_ *W* associated with these estimates. However, close inspection of the data revealed the possibility that a low value (mean Δlog_10_ *W* = −0.698 ± 0.215 standard error of the mean) in the high co-infection group of lineages might be driving this result. To test this possibility, we reanalyzed our data by excluding the value. Results showed that presence or absence of this value did not affect the outcome of our analysis or its conclusions; rather, we still observed a statistically greater variance in fitness change for the high co-infection lineages relative to their low co-infection counterparts (Table S1).

**Table 1:**
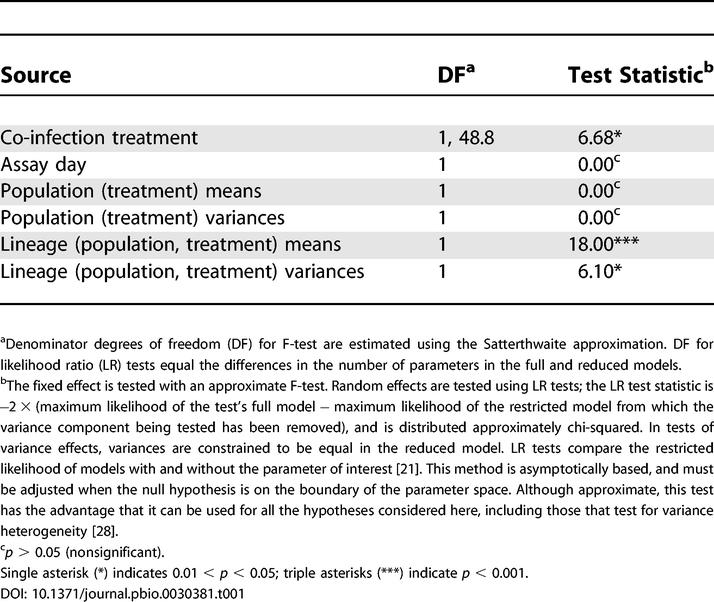
Mixed Linear Models Testing Differences in Mean and Variance of Change in Log_10_ Fitness for Bottlenecked Virus Lineages Differing in Co-Infection History

Our analysis comparing variance in Δlog_10_ *W* between the two groups of evolved lineages is generally regarded as a rigorous method to test for differences in robustness among groups of lineages with differing ecological histories [2,20]. However, some theory also suggests that increased mutational robustness should coincide with reduced effects of mutations on mean fitness [22]. It is widely assumed that the majority of spontaneous mutations are deleterious; thus, mutation accumulation experiments that foster the action of genetic drift over natural selection should on average lead to decreased fitness in a bottlenecked lineage. Our results were consistent with this logic; the grand mean of Δlog_10_ *W* was below zero for both sets of treatment lineages following mutation accumulation (Figure 1). Within each group, at least one-half of the lineages showed mean values below zero, but these were rarely statistically significant, most likely due to the high measurement error expected in RNA systems. Many of the lineages founded by viruses that evolved under low co-infection showed a positive change in fitness, but none of these values was significantly different from zero; thus, we cannot conclude from the data whether the fixed mutation (if a mutation fixed at all) was neutral, mildly deleterious, or mildly beneficial. However, by definition this result is consistent with the relatively greater robustness of low co-infection–evolved viruses, as we would expect phenotypic constancy to cause their Δlog_10_ *W* values to hover close to zero. Also consistent was the greater number of high co-infection lineages experiencing a statistically significant drop in fitness (Table S2), but this difference among groups was minimal (i.e., four versus three significant lineages). More importantly, however, the significantly smaller magnitude of mean Δlog_10_ *W* for the low co-infection lineages (Table 1) was a further indicator that these viruses were more robust than the lineages historically evolved under high co-infection. Once again, we found this conclusion was unaffected when we reanalyzed the data by removing the outlying lineage in the high co-infection group (Table S1).

**Figure 2:**
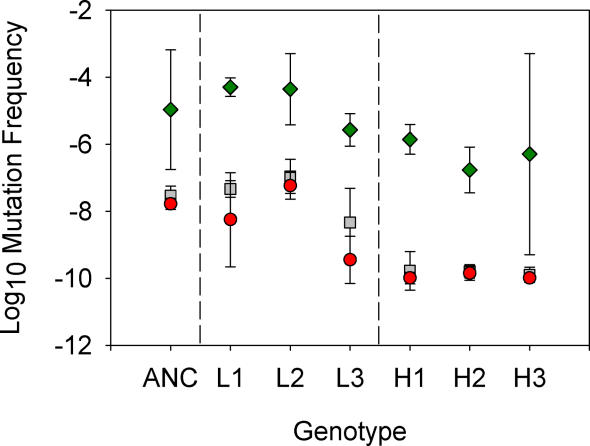
Differences in Virus Robustness Are Not Confounded by Increased Mutation Frequencies in Viruses Evolved under High Co-Infection Mean mutation frequency (± 95% confidence interval) was assayed for wild-type φ6 (ANC), and for one pre-bottleneck clone isolated from each population evolved under low co-infection (L1–L3) and high co-infection (H1–H3). Assays on *P. tomato* (circles) and *P. atrofaciens* (squares) were replicated 5-fold. Assays for *P. atrofaciens* mutants challenged with growth on *P. tomato* (diamonds) were replicated 6-fold, except for H3 (*n* = 2).

Claims of mutational robustness are easily confounded by mutational differences among genotypes [5]. For example, it could be that viruses evolved at low and high levels of co-infection are equally robust, but less frequent mutation of low co-infection lineages is responsible for the lower observed variance in Δlog_10_ *W.* To test this alternative explanation, we measured mutant frequencies (see Materials and Methods) occurring on two novel hosts, *P. syringae* pv.*atrofaciens* and pv. *tomato,* using one pre-bottleneck clone randomly chosen from each population in the two treatments. Host-range mutations on the two hosts occur at different viral loci, meaning that the vast majority of φ6 mutants arising on *P. atrofaciens* cannot infect *P. tomato,* confirmed by genome sequencing of host-range mutants (S. Duffy, C. Burch, and P.E. Turner, unpublished data). The data (Figure 2) did not support the alternative hypothesis because mutant frequencies on each host were not significantly higher for the strains historically evolved under high co-infection. Rather, mutants on *P. atrofaciens* occurred significantly less often for strains evolved under high co-infection (analysis of variance [ANOVA] with *F*_[1,4]_ = 30.49, *p* = 0.0053), and mutant frequencies on *P. tomato* did not differ according to past ecological history (ANOVA with *F*_[1,4]_ = 6.48, *p* = 0.0636). It is unknown whether the frequencies of host-range mutants are representative of mutations occurring elsewhere in the genome, but the robustness differences we observed cannot be alternatively explained by these data.

## Discussion

Mutation and natural selection are cornerstones of evolutionary biology, but elucidating their interplay has proved challenging. To fill this intellectual gap, we conducted experiments to better understand how selection can drive the evolution of genetic robustness. The inherently high error rates in genome replication occurring in RNA viruses make them an obvious choice for examining the adaptive evolution of mutational robustness. We predicted that selection for robustness should be weakened when the RNA phage φ6 experiences high levels of co-infection, owing to virus complementation that buffers mutational effects [10]. Consistent with this hypothesis, our data showed that φ6 genomes evolved under high co-infection were less robust than those propagated under low co-infection, demonstrated by their greater mean magnitude and variance in fitness changes brought on by accumulation of random non-lethal mutations. Our mutation frequency data suggested that the observed difference in robustness was not alternatively explained by elevated mutation frequencies in the viruses evolved under high co-infection.

One possibility is that the rare occurrence of complementation at low co-infection caused these viruses to adapt by evolving greater robustness than the wild-type φ6 ancestor; but this is unknown. If so, our data suggest it is unlikely that this greater robustness was accompanied by more accurate RNA replication; ANOVA shows mutation frequencies of the low co-infection genotypes do not differ from the ancestor on either host (*P. atrofaciens: F*_[1,2]_ = 0.00, *p* = 0.9940; *P. tomato: F*_[1,2]_ = 0.17, *p* = 0.7175). This result is intriguing because the robust viruses seem to feature higher mutation frequencies on average. In turn, the data suggest that evolution of mutational robustness (whatever the underlying molecular mechanism) allows RNA viruses to tolerate less-accurate genome replication, perhaps explaining why these viruses remain highly mutable. Unraveling the exact molecular mechanism(s) for these results would be of great interest. Furthermore, our study implies a need for population genetic models that consider the impact of adaptive robustness on the evolution of replication fidelity, theory that has not been previously explored.

For the wild-type φ6 ancestor and evolved genotypes in our study, mutation frequencies were lower than expected given the ease with which host-range–marked mutants of φ6 were obtained in previous studies. That is, host-range mutants occurred at frequencies ranging between 10^−8^ and 10^−10^ on *P. atrofaciens and P. tomato,* whereas we have observed frequencies between 10^−4^ and 10^−6^ for φ6 on the host *P. pseudoalcaligenes* [10,23]. To explain this disparity, we hypothesized that the host-range phenotypes in the current study resulted from a two-step mutation requiring a precursor allele change. We tested this idea by measuring the frequency with which *P. atrofaciens* mutants were able to further mutate to infect *P. tomato* hosts (see Materials and Methods). The data (Figure 2) supported the assumption that the host-range mutations occur at different loci (i.e., further mutation was needed for growth on the second host); the two-step requirement was also evident because these assays yielded the expected mutant frequencies between 10^−4^ and 10^−6^. Also, these results further suggested that the decreased fitness effects in the low co-infection viruses did not result from more accurate RNA replication, as values for the ancestor and these strains did not differ in the two-step experiment (*P. atrofaciens* mutants challenged with growth on *P. tomato: F*_[1,4.26]_ = 0.07, *p* = 0.8126). Most important, we found that mutation frequency in these assays was significantly lower in the high co-infection viruses (ANOVA: with *F*_[1,4.26]_ = 9.30, *p* = 0.0351), which again argues that our robustness conclusions are not confounded by elevated mutation rates in these viruses. Overall, our data suggest that mutation rate governed by the accuracy of the viral replicase is a trait under selection, especially in the viruses evolved at high co-infection. It is unclear why viruses that frequently experience complementation would evolve higher fidelity of replication, and this may be a pleiotropic effect of selection occurring elsewhere in the genome. The possible adaptive significance of this result is unclear and merits future exploration.

We considered the potential relevance of two other possible confounding factors in our study. First, we examined whether the two groups of viruses differed in fitness prior to mutation accumulation; the alternative explanation is that the low co-infection viruses were already of low fitness (in comparison to the high co-infection strains), and that fixation of one or more additional mutations via bottlenecking did not lead to a further reduction in their fitness. That is, lower fitness genotypes of φ6 are shown to be less affected by addition of further deleterious mutations, a result demonstrating that diminishing-returns epistasis can operate in phage φ6 [19]. This phenomenon would provide an alternative explanation for our data if the average fitness of pre-bottleneck clones drawn from the low co-infection populations was lower than the fitness of those sampled from the high co-infection treatment. We tested this idea by determining whether the fitness of the 60 pre-bottleneck clones in our study differed according to co-infection treatment. Results showed no statistical difference (ANOVA with *F*_[1,3.94]_ = 0.05, *p* = 0.84). Thus, we rejected the possibility that diminishing-returns epistasis, or any argument that hinged on differing fitness among the starting clones, provided an alternative explanation for our results.

We also considered that erroneous conclusions may be drawn from mutation accumulation experiments if conditions impose a change in selective environment for only a subset of test lineages. A similarity between the mutation accumulation experiment and the low MOI treatment is that viruses must infect cells alone, whereas in every fifth generation of the high MOI treatment viruses are forced to undergo co-infection. Thus, it might be argued that the high co-infection lineages (but not low co-infection lines) experienced a change in growth conditions, and that the magnitude and variability in their performance in the bottlenecking experiment results from adaptation to the new conditions. However, we believe that this could not have been a confounding factor in our study for several reasons.

First, the pre-bottleneck clones drawn from both MOI treatments performed equally well prior to mutation accumulation (see above), as measured using fitness assays conducted at low MOI. These data may seem surprising, given that at generation 200 the treatment populations showed significantly different fitness under low-MOI conditions [14]. However, this difference was at the level of populations, whereas the current study used genotypes drawn from these populations; mean fitness of a microbial population can differ from mean fitness of clones drawn from the population [24,25]. One possibility is that the parent population contains variants of very high fitness in the assay environment. Because fitness assays in microbial systems typically span multiple generations (i.e., five generations in our case), a genetic variant within the mixed population will be overrepresented by the end of the assay, which elevates the overall performance of the population revealing its “evolutionary potential” in the assay environment. In contrast, a clone's performance in two habitats reveals only its phenotypic plasticity and not the evolutionary potential of its parent population. This could easily account for the very large fitness differences at low MOI observed for populations evolved under low and high co-infection, contrasted with the equal fitness of pre-bottleneck clones drawn from these populations assayed in the same environment.

Second, the mutation accumulation experimental conditions can be considered novel for all test lineages. In particular, the experimental evolution necessitated that viruses attached to host cells in liquid medium prior to overnight growth on agar plates, whereas the mutation accumulation habitat did not. Even if phage φ6 attachment to cells in liquid versus on agar surfaces is fundamentally the same (this is not well described), it is plausible that these two habitats differ substantially in the dispersal of phages between infected cells (T. Berngruber and L. Chao, unpublished data).

Third, it is highly unlikely that the test lineages could have adapted to the mutation accumulation conditions at all. Plaque formation on agar necessarily involves expansion of the bottlenecked population to large size, which allows for some positive selection. That is, a plaque forms from a single virus experiencing five generations of growth on the plate, where the average number of progeny made by an infecting virus is 100 particles, yielding ~10^8^ virus particles within a plaque. However, the mutation accumulation design prevents virus adaptation because the action of genetic drift overwhelms that of natural selection; i.e., lineages evolve at an effective population size of *N* ≍ 2, the harmonic mean of the serial passage fluctuating between 1 and 10^8^ viruses.

Our study suggests several intriguing possibilities for future research in φ6 and other RNA viruses. Identifying whether the low co-infection populations, the high co-infection populations, or both have changed in robustness relative to their common ancestor could help shed light on the genetic mechanism(s) underlying this difference in the φ6-derived viruses. (Our goal was to examine the impact of co-infection history on evolution of robustness, which was achieved by comparing effects of mutation accumulation among lineages founded by random clones drawn from each treatment, so our study omitted lineages founded by the ancestor because these would lack standing genetic variation, preventing any direct comparisons to results from the evolved groups.) The 300-generation experiment [14] that preceded mutation accumulation was probably too short for evolution of de novo adaptive epistatic interactions [26]. Therefore, it is perhaps more likely that the high co-infection populations became less robust (relative to the ancestral state) due to weakened selection for robustness owing to genetic buffering provided by complementation. Future viral-genomic research could determine whether this outcome occurred due to fixation of non-adaptive alleles, or due to antagonistic pleiotropy (i.e., selection for alleles that enhance virus fitness under co-infection, but diminish performance in the unselected trait robustness). Recent studies have addressed the evolutionary consequences of co-infection, especially in RNA viruses [9–11]. During co-infection, viruses can be considered analogous to polyploid organisms because several copies of the virus genome are present inside the same host cell. Co-infection may be advantageous in the short term because it allows for complementation, which can partially or fully mask the cost of deleterious mutations. However, co-infection may be detrimental in the long term, because complementation can slow the rate at which deleterious alleles are eliminated from the virus population [10] and can promote selection for defective-interfering particles or other cheater genotypes that reduce mean population fitness [16]. Our experiments suggest that an additional cost of co-infection is the weakened selection for robustness that may cause evolution of relatively brittle genomes. We obtained this result by contrasting the strength of selection for adaptive robustness in low versus high co-infection populations. In this way, we purposefully avoided the more difficult task of examining de novo evolution of robustness that current theory suggests is most likely to occur after populations have reached equilibrium; other researchers might benefit from such an approach in their study systems.

## Materials and Methods

### Strains and culture conditions

Phages and bacteria were cultured at 25 °C in LC medium, Luria-Bertani broth (pH 7.5) [14]. Overnight cultures of bacteria were grown from a single colony placed in 10 ml LC medium, with shaking incubation (120 rpm). *P. phaseolicola* was purchased from American Type Culture Collection ( ATCC #21781; Manassas, Virginia, United States). L. Mindich (Public Health Research Institute, Newark, New Jersey, United States) kindly provided host strain *P. pseudoalcaligenes* ERA (East River isolate A); G. Martin (Cornell University, Ithaca, New York, United States) kindly provided*P. syringae* pv.*tomato* and *P. syringae* pv.*atrofaciens*. Bacterial stocks were stored in 4:6 glycerol/LC (v/v) at −80 °C. Viruses were grown on lawns made from overnight bacterial cultures. Agar concentrations in plates were 1.5% and 0.7% for bottom and top LC agar, respectively. Plates contained 3 ml of top agar and a 200 μl bacterial lawn. Phage lysates were prepared by growing viruses on a *P. phaseolicola* lawn for 24 h; plaques were then collected and filtered (0.22 μm filter, Durapore; Millipore, Billerica, Massachusetts, United States) to remove bacteria. Phage lysates were stored at −20 °C in 4:6 glycerol/LC (v/v).

### Virus evolution

Virus populations were experimentally evolved at MOI of 0.002 or 5 for 250 generations [14], followed by an additional 50 generations in our laboratory. MOI was imposed every fifth generation by allowing viruses to attach to *P. phaseolicola* cells in a test tube containing liquid LC medium, incubated for 40 min at 25 °C. These infected cells were then diluted and plated on a *P. phaseolicola* lawn on which viruses form distinct plaques. Population size of *N* ≍ 500 was held constant across treatment populations by controlling the number of harvested plaques [14]. On average, each plaque in the low co-infection treatment was produced by one phage, whereas that in the high co-infection treatment was produced by five co-infecting parents; thus, *N* ≍ 500 was controlled by harvesting 500 plaques from a low MOI population and 100 plaques from a high MOI population. After the experimental evolution, ten clones were chosen at random from each population. Each of the 60 clones was then used to found a single lineage subjected to 20 d of mutation accumulation via bottlenecking [17,19]. Bottlenecking was achieved by cutting out a single 24-h-old plaque from a *P. phaseolicola* lawn, placing it in sterile liquid medium, and vortexing gently to disperse the progeny viruses contained within the plaque. A sterile platinum loop was then used to streak the viruses onto agar containing a naive host lawn. After 24-h incubation, the viruses formed distinct plaques, and the process was repeated for 20 consecutive days. Because each plaque grows from a single virus, daily passage caused a test lineage to be forced through an extreme bottleneck (population size of one individual), in which the intense drift allows fixation of non-lethal mutations, the majority of which are presumed to be deleterious. Because the phage expand to approximately 10^8^ phage in five generations within a plaque, this creates the opportunity for selection to operate within a population propagated by such one-plaque transfers. Thus, positive selection on the plate should bias against fixation of highly deleterious mutations, and it is possible to observe the fixation of a rare beneficial mutation in a bottlenecked lineage [17]. However, the intensity of selection is not sufficient to overcome the intense genetic drift generated by the bottlenecks, causing fitness to generally decline due to accumulation of deleterious mutations [17–19]. Also, we note that in our experiments any confounding effects of co-infection on the plate could be ignored because the virus dilution (or streak) grew within a superabundant lawn (i.e., no more than 400 viral plaques were allowed to form on a *P. phaseolicola* lawn containing 8 × 10^8^ cells; MOI on the plate of 5 × 10^−7^).

### Fitness assays

Fitness assays consisted of paired-growth experiments [17], which compared 24-h growth on *P. phaseolicola* of a focal genotype relative to a common competitor of φ6 bearing a genetic marker (i.e., ability to infect *P. pseudoalcaligenes* bacteria [27]). The competitors were mixed at a 1:1 volumetric ratio, and then a dilution of this mixture containing approximately 400 viruses was plated on a *P. phaseolicola* lawn. Because no pre-attachment occurred before plating, every virus in the lawn infected a cell alone. After 24-h incubation, the approximately 400 plaques were harvested and filtered to obtain a cell-free lysate. The ratios of competing genotypes in the starting mixture (*R*_0_) and in the harvested lysate (*R*_1_) were obtained by plating on mixed lawns of *P. phaseolicola* and *P. pseudoalcaligenes* (200:1 mixture) on which ordinary and host-range genotypes form turbid and clear plaques, respectively. Fitness *(W)* was defined as the relative change in ratio of ordinary to host-range virus, or *W* =*R*_1_ */R*_0_ *.*

### Mutant frequency estimates

We measured the appearance of host-range mutants formed on selective plates: bacterial lawns of *P. tomato* or *P. atrofaciens*. A high-titer lysate (typically ~10^10^ viruses per ml) of a virus genotype was grown and titered on *P. phaseolicola,* and a sample of the lysate was then screened on a selective plate. Mutant frequency was calculated as the number of plaque-forming mutants per viruses in the inoculum; when no mutants were found, we used the limit of detection as a conservative estimate. Acquisition of a second host-range mutation was examined by rearing phage mutants on *P. atrofaciens,* followed by selective plating on *P. tomato*.

### Statistical analyses

A mixed general linear model controlling for experimental assay day was run [21], testing the effects of the fixed-factor co-infection treatment (low MOI or high MOI) and the random-factors population nested within treatment, and the lineage nested within population and treatment on the difference in the (log-transformed) Wrightian relative fitnesses of the founding and final clone from each bottleneck lineage. For these two random factors, a term testing both for variability in means among levels and for differences in variance between the treatments was included. For example, population (treatment) means is a term testing for variability in means among populations, whereas population (treatment) variances is a term testing for whether low-MOI populations and high-MOI populations differ in variance. The fixed effect was tested using an approximate F-test with the denominator degrees of freedom estimated using the Satterthwaite approximation. These denominator degrees-of-freedom estimates depend both on sample sizes and variance structure. Random factors were tested using likelihood-ratio tests, which compare the restricted likelihood of models with and without the parameter of interest [21]. This method is asymptotically based and must be adjusted when the null hypothesis is on the boundary of the parameter space. Although approximate, this test has the advantage that it can be used for all the hypotheses considered here, including those that test for variance heterogeneity [28].

## Supporting Information

Table S1 Summary of Changes in Log_10_ Fitness for Bottlenecked Virus Lineages (91 KB DOC).

Table S2 Reanalysis of Mixed Linear Models Shown in Table 1, Excluding an Unusually Low Value (Population H2, Lineage 5) (26 KB DOC).

## Abbreviations

ANOVA: analysis of variance
MOI: multiplicity of infection
pv: pathovar
