# Supplementary material for: Evolution of Mutational Robustness in an RNA Virus
Source: PLoS Biol. 2005 Nov 1;3(11):e381. doi: 10.1371/journal.pbio.0030381 (PMC1275523; doi:10.1371/journal.pbio.0030381)
Supplement: Supplementary file 1 [file pbio.0030381.st001.doc]

Table S1. Summary of Changes in Log_10_ Fitness for Bottlenecked Virus Lineages

| Low Co-infection Populations | | | | | High Co-infection Populations | | | | |
| --- | --- | --- | --- | --- | --- | --- | --- | --- | --- |
| Population | Lineage | ∆log W^a^ | s.e.m. | *P* | Population | Lineage | ∆log W^a^ | s.e.m. | *P* |
| 1 | 1 | -0.079 | 0.161 | 0.670 | 1 | 1 | -0.339 | 0.086 | 0.059 |
| 1 | 2 | 0.047 | 0.118 | 0.730 | 1 | 2 | -0.161 | 0.040 | 0.056 |
| 1 | 3 | -0.125 | 0.120 | 0.409 | 1 | 3 | -0.105 | 0.035 | 0.097 |
| 1 | 4 | -0.045 | 0.084 | 0.649 | 1 | 4 | -0.316 | 0.140 | 0.152 |
| 1 | 5 | 0.003 | 0.070 | 0.972 | 1 | 5 | -0.053 | 0.028 | 0.198 |
| 1 | 6 | 0.020 | 0.112 | 0.872 | 1 | 6 | -0.030 | 0.023 | 0.334 |
| 1 | 7 | 0.088 | 0.193 | 0.691 | 1 | 7 | **-0.232** | 0.033 | 0.019 |
| 1 | 8 | **-0.195** | 0.006 | 0.001 | 1 | 8 | -0.085 | 0.087 | 0.430 |
| 1 | 9 | -0.172 | 0.124 | 0.299 | 1 | 9 | 0.085 | 0.052 | 0.245 |
| 1 | 10 | 0.042 | 0.048 | 0.470 | 1 | 10 | 0.127 | 0.055 | 0.146 |
| 2 | 1 | 0.038 | 0.131 | 0.802 | 2 | 1 | -0.044 | 0.052 | 0.489 |
| 2 | 2 | 0.018 | 0.066 | 0.806 | 2 | 2 | 0.125 | 0.042 | 0.096 |
| 2 | 3 | 0.122 | 0.096 | 0.333 | 2 | 3 | -0.142 | 0.081 | 0.222 |
| 2 | 4 | **-0.217** | 0.049 | 0.047 | 2 | 4 | -0.070 | 0.103 | 0.565 |
| 2 | 5 | 0.092 | 0.026 | 0.072 | 2 | 5 | -0.698 | 0.215 | 0.083 |
| 2 | 6 | **-0.353** | 0.015 | 0.002 | 2 | 6 | -0.065 | 0.138 | 0.686 |
| 2 | 7 | 0.217 | 0.096 | 0.151 | 2 | 7 | **0.076** | 0.014 | 0.033 |
| 2 | 8 | 0.018 | 0.015 | 0.341 | 2 | 8 | -0.054 | 0.029 | 0.206 |
| 2 | 9 | -0.024 | 0.139 | 0.879 | 2 | 9 | -0.350 | 0.112 | 0.088 |
| 2 | 10 | 0.008 | 0.057 | 0.902 | 2 | 10 | **-0.338** | 0.057 | 0.028 |
| 3 | 1 | -0.048 | 0.037 | 0.332 | 3 | 1 | -0.148 | 0.060 | 0.134 |
| 3 | 2 | -0.055 | 0.081 | 0.567 | 3 | 2 | -0.074 | 0.150 | 0.671 |
| 3 | 3 | -0.024 | 0.087 | 0.811 | 3 | 3 | **-0.471** | 0.099 | 0.042 |
| 3 | 4 | -0.074 | 0.045 | 0.239 | 3 | 4 | -0.234 | 0.155 | 0.271 |
| 3 | 5 | -0.060 | 0.068 | 0.474 | 3 | 5 | -0.017 | 0.048 | 0.757 |
| 3 | 6 | -0.131 | 0.148 | 0.470 | 3 | 6 | -0.037 | 0.088 | 0.713 |
| 3 | 7 | 0.032 | 0.115 | 0.808 | 3 | 7 | 0.001 | 0.051 | 0.989 |
| 3 | 8 | -0.102 | 0.109 | 0.445 | 3 | 8 | -0.160 | 0.182 | 0.472 |
| 3 | 9 | 0.074 | 0.086 | 0.480 | 3 | 9 | -0.125 | 0.049 | 0.126 |
| 3 | 10 | 0.092 | 0.081 | 0.375 | 3 | 10 | 0.075 | 0.135 | 0.633 |

^a^Mean of *n* = 3 independent measurements; values in bold significantly differ from zero (*P* < 0.05).
