# Supplementary material for: Evolution of Mutational Robustness in an RNA Virus
Source: PLoS Biol. 2005 Nov 1;3(11):e381. doi: 10.1371/journal.pbio.0030381 (PMC1275523; doi:10.1371/journal.pbio.0030381)
Supplement: Supplementary file 2 [file pbio.0030381.st002.doc]

Table S2. Reanalysis of Mixed Linear Models Shown in Table 1, Excluding an Unusually Low Value (Population H2, Lineage 5)

Source	DF^a^	Test Statistic^b^

Co-infection Treatment	1, 51.7	5.59*

Assay Day	1	0.00^NS^

Population (treatment) [means]	1	0.00^NS^

Population (treatment) [variances]	1	0.00^NS^

Lineage (population, treatment) [means]	1	10.8**

Lineage (population, treatment) [variances]	1	12.9**

^a^Denominator DF (degrees of freedom) for F-test is estimated using the Satterthwaite approximation. DF for likelihood ratio (LR) tests equals the difference in the number of parameters in the full and reduced models.

^b^The fixed effect is tested with an approximate F-test. Random effects are tested using LR tests; the LR test statistic is –2 × (maximum likelihood of the test’s full model – maximum likelihood of the restricted model from which the variance component being tested has been removed), and is distributed approximately chi-squared. In tests of variance effects, variances are constrained to be equal in the reduced model. LR tests compare the restricted likelihood of models with and without the parameter of interest [21]. This method is asymptotically based, and must be adjusted when the null hypothesis is on the boundary of the parameter space. Although approximate, this test has the advantage that it can be used for all the hypotheses considered here, including those that test for variance heterogeneity [28]. ^NS^, p > 0.05; *, 0.01 < p < 0.05; **, 0.001<p<0.05.
